# Real-Time Safety Risk Assessment Based on a Real-Time Location System for Hydropower Construction Sites

**DOI:** 10.1155/2014/235970

**Published:** 2014-07-09

**Authors:** Hanchen Jiang, Peng Lin, Qixiang Fan, Maoshan Qiang

**Affiliations:** ^1^State Key Laboratory of Hydroscience and Engineering, Tsinghua University, Haidian, Beijing 100084, China; ^2^China Yangtze Three Gorges Group Corporation, Beijing 100038, China

## Abstract

The concern for workers' safety in construction industry is reflected in many studies focusing on static safety risk identification and assessment. However, studies on real-time safety risk assessment aimed at reducing uncertainty and supporting quick response are rare. A method for real-time safety risk assessment (RTSRA) to implement a dynamic evaluation of worker safety states on construction site has been proposed in this paper. The method provides construction managers who are in charge of safety with more abundant information to reduce the uncertainty of the site. A quantitative calculation formula, integrating the influence of static and dynamic hazards and that of safety supervisors, is established to link the safety risk of workers with the locations of on-site assets. By employing the hidden Markov model (HMM), the RTSRA provides a mechanism for processing location data provided by the real-time location system (RTLS) and analyzing the probability distributions of different states in terms of false positives and negatives. Simulation analysis demonstrated the logic of the proposed method and how it works. Application case shows that the proposed RTSRA is both feasible and effective in managing construction project safety concerns.

## 1. Introduction

The construction industry is one of the most dangerous industrial sectors worldwide [1, 2]. The working environment and the work tasks themselves are complex. The number of workers on site generally is large. Heavy machinery and a multitude of pipes, materials, and cables are always in evidence. In addition, sites are frequently not “tidy”; it is not surprising that both the fatality and badly injured accident rates on construction site are higher than those in other areas of work [3, 4]. According to the Bureau of Labor statistics in the United States [5], 4253 construction workers died on sites between 2008 and 2012. In China, the official statistics show that, in the one year, 2007, construction industrial accidents caused the deaths of 2722 workers [6]. Hydropower projects, in particular, have been found to have tougher working conditions than other construction sites and safety management of such construction projects is more difficult than that in any other segment of the industry [7]. Currently, China has been continuously developing large hydropower projects on main rivers, such as the Jinsha, Nujiang, and Yarlung Tsangpo River, investing large financial sums and manpower resources [8, 9]. Hence, without the improvement of the safety management, culture of the construction industry and the implementation of the national policy, which is “safety first and prevention oriented” [10] and which is also a vital management goal of hydropower enterprises in China, will not be achieved and it is unlikely that the accident rate on construction sites will decrease.

Recent studies related to construction safety management have asserted that most accidents on sites could have been reduced and some even eliminated, if there existed an effective and consistent safety management process of identification, planning, education/training, and inspection [11]. Safety risk assessment is very important for developing a safety management system [12–14]. Numerous risk assessment methods, such as safety checklists, fault tree analysis (FTA), and likelihood exposure consequences (LEC), have been developed to assist construction engineers and project managers in safety risk management [15]. The LEC method is widely used in casualty risk assessment in industrial construction [16]. Risk is considered to be the product of three factors: risk likelihood/probability (*L*), risk exposure frequency (*E*), and consequences (*C*). Casanovas et al. [17] presented a risk index which included 19 construction work activities and used the product of consequence and probability to obtain the risk value of each activity. In addition, accident statistics, event tree analysis, and failure mode and effects analysis are also applied in safety management in the construction industry [18]. All the above methods, however, produce qualitative or semiqualitative analysis results. Such results cannot be considered reliable. For example, all of the three factors of LEC method are based on the subjective evaluation of experts. Differences in the experience of different experts may lead to different evaluation results of the same risk [16]. In addition, most of these methods are passive approaches, heavily dependent on historical data and lacking timeliness [17, 19]. As a consequence, these methods, although producing results of interest and for guidance, cannot be used to quantitatively assess the current status of safety performance and the risk value.

In order to operate a safety risk assessment procedure accurately, some quantitative methods based on probability theory and fuzzy sets theory are proposed. Using machine learning algorithms, Matías et al. [20] performed the identification of hazardous sources on construction sites and the subsequent determination of the relationship between the accident and the cause. Matías et al. [21] designed a Bayesian network (BN) based method designed to analyze workplace accidents caused by falls from heights. Leu and Chang [22] successfully combined probabilistic methods with traditional methods (event tree analysis), enabling the setting up of a complete logic model to build a quantitative risk assessment method for construction projects. Regarding fuzzy set theory, Lee and Halpin [23] proposed a quantitative approach based on fuzzy logic for measuring the effects of accidents. Nunes [24] developed an expert system aimed at supporting risk analysis based on fuzzy sets. Azadeh et al. [25] developed a fuzzy expert system for performance assessment of health, safety, environment, and ergonomic system factors in a gas refinery with the objectives of the reduction of human error, creation of expert knowledge, and interpretation of large amounts of vague data. These quantitative methods based on probability and fuzzy sets theory offer relatively accurate evaluation results compared to those of the traditional methods and have been implemented in practical projects. Nevertheless, none of the above methods have been able to adapt to the dynamic context of construction projects, particularly those complex activities which contribute greatly to the safety risk of on-site workers. In addition, such methods cannot provide real-time information about safety states, which is a necessity to enable timely response and rescue.

Recently, extensive studies of real-time risk assessment have been carried out. An example is provided by the hidden Markov model (HMM) which is effective in many temporal and real-time pattern recognition applications, such as human action recognition [26, 27], speech recognition [28], and prediction of unknown events [29, 30]. Real-time risk assessment methods based on the HMM focus on the field of network security. These methods use the HMM to process the observation symbol sequences of invasions and attacks to determine the real-time safety risk of a network [31–33]. In principle, the HMM based risk assessment method is accessible, but real-time safety risk assessment studies in construction management have not been found in the literature. The main reason for this could be the lack of availability of on-site asset real-time location information.

As a consequence, some studies have focused on the development of intelligent control systems [34–37], adopting advanced communication technologies [38, 39], real-time location [40, 41], and intelligent algorithms as the means of developing new automated management and monitoring systems [42–44]. However, these particular studies concentrate more strongly on technical performances, such as accuracy of positioning and optimization of communication frequency, while significant features such as safety management functions are not directly targeted. Geofencing which deals with the automatic detection of the entrance of individuals into dangerous preset forbidden zones is the basic safety management model revealed in the literature [39, 40]. But the simple trigger logic of the geofencing can only offer managers poor binary information concerning on-site workers' behaviour (either within the forbidden zones or not in the forbidden zones) and may lead to frequent false positives. As a consequence, the development of an integrated method to perform real-time safety risk assessment of on-site workers based on probability concepts is necessary. The workers' real-time location data should be provided by real-time location technologies. Overall, the development of automatic monitoring technology, especially the real-time location technology for individuals, provides great feasibility for implementation of real-time safety risk assessment. And the real-time location system for personnel and the monitoring system for structure constitute a complete solution for health and safety management in industrial construction.

In this study, GPS and Wi-Fi locating technology were chosen as the fundamental tracking tools. HMM was used as the risk assessment algorithm in constructing a RTSRA method for the safety management of large hydropower construction projects. The rest of this paper is organized as follows.  Section 2 describes the construction of an assessment framework, including that of the real-time tracking system and the HMM modelling process. In Section 3, a simulation analysis is carried out on safety risk assessment process.  Section 4 gives a preliminary application case of a dam construction site.

## 2. Risk Assessment Framework of RTSRA

**Figure 1:**
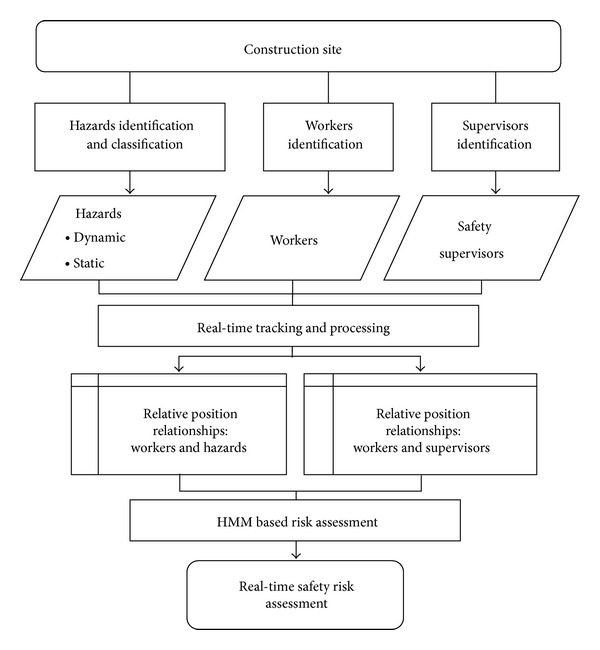
Logic of the real-time safety assessment method.

Figure 1 shows the RTSRA logic diagram. The construction site is the environment in which the safety risk assessment is conducted. On-site assets affecting the safety risk are catalogued into the three groups: workers, hazards including dynamic and static hazards, and safety supervisors. By employing the RTLS, site assets location data are collected. Relative position relationships, in the form of a time series, between workers and hazards as well as for safety supervisors are obtained by processing and analysing the location data. The HMM is used to find the most likely probability distribution of each monitored worker's states, followed by obtaining the real-time safety risk for each worker.

RTSRA, differing significantly as regards assessment objects found in earlier studies, focuses on specific worker real-time risks rather than risks of general on-site activities. Thus, RTSRA can be classified as a Human-Centered method [45]. Safety risk assessment performs as a vital potential precursor to a tool to serve on-site workers, while the Human-Centered method of RTSRA has a more direct objective in which it gives an integrated assessment of risks associated with various hazards on construction site.

**Figure 2:**
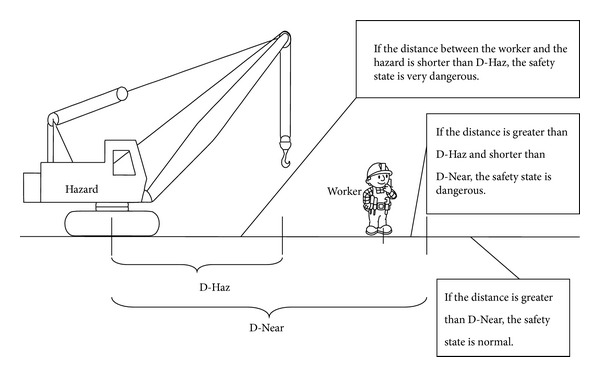
Definition of safety states associated with a crane.

On site workers carry smart phones as mobile targets implementing the provision of monitoring and communication enabled by a customized Android App. Thus, RTSRA not only is able to provide worker real-time safety risks information for managers but also can send warning or “alert” short messages to workers and site supervisors. The RTSRA enables computing to be bidirectional and ubiquitous by utilizing state-of-the-art computer and communication technology. Based on the relative positions between workers and hazards, different safety states are defined.  Figure 2 gives an example of safety states definition associated with a crane. The HMM calculates the probabilities of different safety states for a worker. The degrees of severities of the different states are obtained from a knowledge base subsystem which extensively explores historical data and expert assessments. The real-time risk *R*_*t*_^*w*^ for a specific worker *w* at time *t* is thus quantified by the following equation [33]:

(1) $$\begin{array}{c} R_{t}^{w}=\overset{N}{\underset{i=1}{\sum }}\;\overset{M_{i}}{\underset{j=1}{\sum }}p_{t}^{w}\left(i,j\right)\times C\left(i,j\right), \end{array}$$

where *p*_*t*_^*w*^(*i*, *j*) is the probability that the worker *w* is in safety state *j* associated with hazard *i* at time *t*, *C*(*i*, *j*) is the cost value (severity) of state *j* associated with hazard *i*, *N* is the number of hazards, and *M*_*i*_ is the number of safety states associated with hazard *i*.

### 2.1. Real-Time Location System

**Figure 3:**
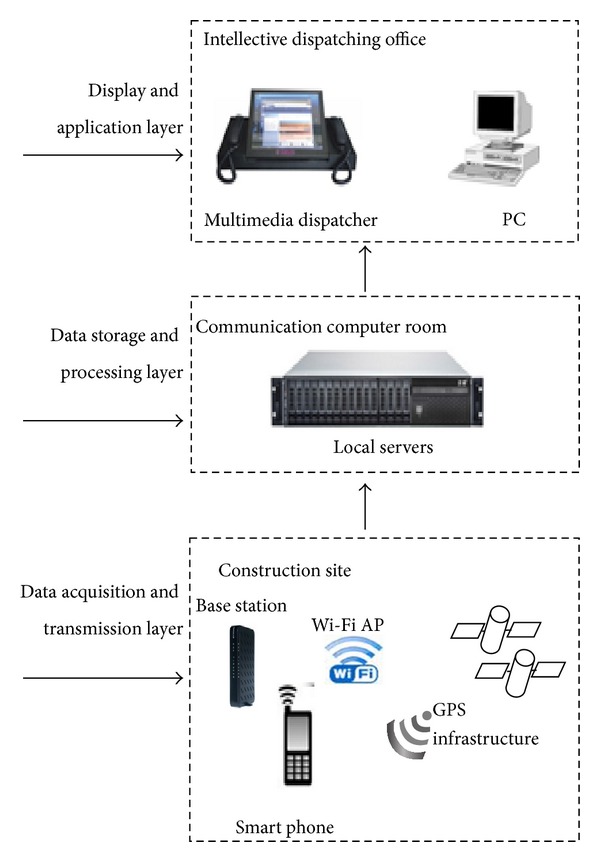
Block diagram of the real-time location system.

This study involved an integrated RTLS [8, 46], which tracked worker and asset locations using GPS and Wi-Fi locating technology. The RTLS framework is divided into the three layers (Figure 3), such as a physical layer where data acquisition and transmission are performed, a middleware layer where data storage is organized and all the location data is processed using a calculating engine, and an application layer providing an interface with end user by various means such as the WEB and mobile phone apps.

The physical layer is the infrastructure required to gather location data. This is composed of smart phones, Wi-Fi router, 3G data network, and optical network. The smart phone works as an RTLS agent with built-in GPS and Wi-Fi modules and has the customized app installed on it. Its function is to receive signals, including GPS signals and Wi-Fi signals, and to upload these digitalized data to the server at fixed time intervals and to calculate current locations using the trilateration algorithm [38]. The network infrastructure includes 3G base stations and a central network for data communication. Wi-Fi access points (APs) are provided as reference points in those indoor areas where GPS signals are not available and GPS infrastructure was used for outdoor positioning. The middleware layer consists of several servers and software systems, which store and retrieve location data, matches and updates the locations on the construction site map, calculates and adjusts location data, and performs other management functions such as data maintenance, load balancing, and system log management. The application layer consists of a series of human machine interfaces (HMIs) for end user. Those compatible display terminals include multimedia dispatchers, personal computers, and portable devices such as smart phones and tablet computers. The main function of this layer is to provide engineers and project managers with visual vision including the real-time trajectories of workers on site, information on work context awareness, and real-time safety risk assessment.

The users of the system can be divided into the two groups of on-site users and off-site users. They access different software as their needs are different. On-site users include workers, site supervisors, and machinery. The software is an Android App which controls the GPS module and Wi-Fi module of the smart phones. GPS is used when users are outdoors and Wi-Fi when users are in indoor places such as tunnels. Off-site users include engineers, project managers, and researchers. The main application for off-site users is a web-based software system allowing access to any authenticated user with an internet access, no matter where they are. The web page provides users with a view of the real-time locations of the on-site assets. The historical trajectories are also available for ad hoc queries. These functions assist engineers and project managers to be aware of the situation on site very conveniently. Moreover, on top of the WEB GIS system, for safety management purposes, the identification and classification of hazards and real-time worker risk assessments are implemented. Thus, a knowledge-based system is created in which system parameters, threshold values, and rules are stored and organized.

### 2.2. Measurable Factors

Unsafe worker activities are direct causes of accidents. Different unsafe activities are usually associated with specific hazards. It has been concluded by Casanovas et al. [17] that there are 19 health and safety risks found in construction work associated with different activities. According to hazard kinetic characteristics, these risks can be divided into dynamic and static groups (Table 1).

Most dynamic hazards on the construction site are caused by vehicles, machinery, and risky tools. A static hazard and its affected area remain unchanged for a relatively long period. These latter hazards consist of elevation changes, hazardous installations, and other dangerous zones. For both dynamic and static hazards, the critical measurable factor is the distance between the workers and hazards [17]. Accidents are more likely to happen, if workers are close to hazards. A dissimilarity is that the position of a static hazard is fixed while a dynamic hazard is always moving. In consequence, in addition to the tracking of workers, dynamic hazards must also be monitored by the RTLS in order to make valid safety risk assessments.

**Figure 4:**
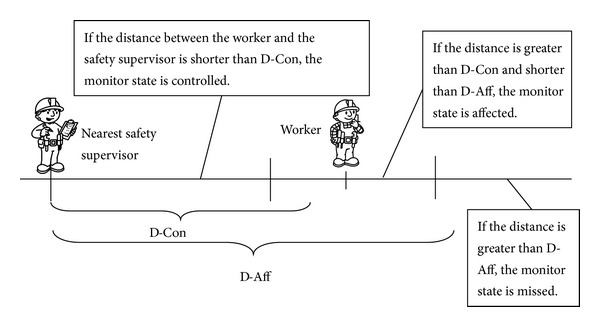
Definition of monitor states with safety supervisor.

**Figure 5:**
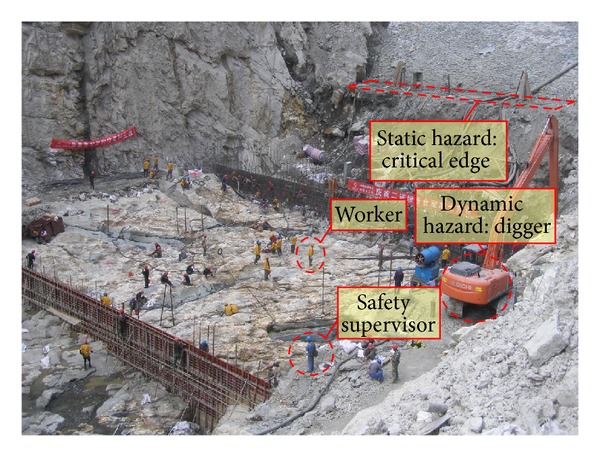
Monitored objects in terms of safety risk assessment on a typical hydropower construction site.

Another critical factor is the presence of safety supervisors. They are responsible for safety management and possess specialized safety knowledge [47]. Effective safety supervision improves the safety performance of workers. The safety supervisor must be in such a position that he can have a positive impact on a worker behaviour [48]. The distance between worker and safety supervisor can be obtained from the RTLS and hence is another measurable factor. Monitor states are compared with safety states in assessing the effectiveness of supervision, as shown in Figure 4. So far, the monitored objects factored into the assessment model include workers, safety supervisors, and dynamic hazards. The locations of static hazards are determined and updated manually.  Figure 5 presents these monitored objects associated with the safety risk assessment in a typical hydropower construction site.

Considering the positive impacts due to the presence of safety supervisors, a reduction factor is added to (1), as follows:

(2) $$\begin{array}{c} R_{t}^{w}=D_{t}^{w}\times \overset{N}{\underset{i=1}{\sum }}\;\overset{M_{i}}{\underset{j=1}{\sum }}p_{t}^{w}\left(i,j\right)\times C\left(i,j\right), \end{array}$$

where *D*_*t*_^*w*^ is the reduction factor associated with the presence of a safety supervisor near worker *w* at time *t*. Other components of (2) have the same meaning as in (1). *D*_*t*_^*w*^ is derived by the following equation:

(3) $$\begin{array}{c} D_{t}^{w}=\overset{L}{\underset{k=1}{\sum }}b_{t}^{w}\left(k\right)\times F\left(k\right), \end{array}$$

where *b*_*t*_^*w*^(*k*) is the probability that the worker *w* is in monitor state *k* at time *t*. *F*(*k*) is the reduction in value of the monitor state *k* and is determined using historical data or by experts.

Comparing (1), (2), and (3), it can be concluded that the calculation of *D*_*t*_^*w*^ is of the same form as a single hazard risk analysis. The key risk assessment process is application of the algorithm determining the probability distributions of safety states and monitor states of a worker.  Section 2.3 describes how to calculate real-time safety state probability distribution associated with a single hazard on the basis of the hidden Markov model.

### 2.3. Modeling Workers as Hidden Markov Model

A HMM is a statistical Markov model in which the system being modeled is assumed to be a Markov process with hidden states [49]. The hidden states are not directly visible, but output dependent on the hidden state is visible. Each hidden state has a probability over the possible out symbol. Hence, the sequence of observation symbols generated by an HMM gives some information about the sequence of hidden states.

To be able to perform real-time risk assessment on a construction site, an RTLS must exist. Positions of workers, hazards, and safety supervisors are all known. Let *W* = {*w*_1_, *w*_2_,…} be the set of workers monitored by the RTLS. To describe the safety state of each worker, discrete-time Markov chains are used. In the simplest case, consider that there is only one hazard on the site. Assume that each worker can be in one of *M* safety states with that only hazard. And the safety states are denoted as *S* = {*s*_1_, *s*_2_,…, *s*_*M*_}. The safety state of a worker changes over time between the states in *S*. The sequence of states through which the worker moves is denoted as *X* = {*x*_1_, *x*_2_,…, *x*_*T*_}, where *x*_*t*_ ∈ *S* is the safety state at time *t*. For the purpose of safety risk assessment, it is assumed that the safety state space consists of the three states, normal (*N*), dangerous (*D*), and very dangerous (*V*); that is, *S* = {*N*, *D*, *V*}. State *N* means safe working and is not subject to any potential safety risk. As a worker gets closer to a hazard, his safety state will move to *D*. A worker in state *D* is subject to an approaching risk, possibly affecting his safety related behaviour. Finally, a worker enters state *V* if he is within the risk's hazardous radius. A worker in state *V* is assumed to be in great danger of serious harm. But in fact, the real safety state of a worker is unknown. It can only be implied by the observation symbol.

The observed positions and relative position relationships are provided by the RTLS. But due to the measurement error of GPS and Wi-Fi locating, there is no one-to-one correspondence relationship between real state and observation symbol. An observation symbol can consist of any of the symbol spaces *O* = {*o*_1_, *o*_2_,…, *o*_*K*_}. These symbols may be used to represent different types of position relationships between workers and hazards, such as very close, close, normal, and far away. The sequence of observation symbols that the system receives is denoted as *Y* = {*y*_1_, *y*_2_,…, *y*_*T*_}, where *y*_*t*_ ∈ *O* is the observation symbol received at time *t*. Based on the sequence of observation symbols, the system performs risk assessment dynamically.

The transition between worker safety states can be represented by a HMM, defined by *λ* = {**Ρ**, **Q**, *π*}, where **Ρ** = {*p*_*ij*_} is the state transition probability distribution matrix for worker *w*, where *p*_*ij*_ = *P*(*x*_*t*+1_ = *s*_*j*_∣*x*_*t*_ = *s*_*i*_), 1 ≤ *i*, *j* ≤ *M*. Hence, *p*_*ij*_ represents the probability that worker *w* will transfer into state *s*_*j*_, given that his current state is *s*_*i*_. In order to estimate the value of **Ρ** for real working conditions, either accident statistical data, known industrial standards, or the subjective opinion of experts must be studied. Machine learning algorithms may be implemented to provide better estimates of **Ρ** over time for the type of site in question. **Q** = {*q*_*j*_(*l*)} is the observation symbol probability distribution matrix for worker *w* in state *s*_*j*_, whose elements are *q*_*j*_(*l*) = *P*(*y*_*t*_ = *o*_*l*_∣*x*_*t*_ = *s*_*j*_), 1 ≤ *j* ≤ *M*, 1 ≤ *l* ≤ *K*. In the model, the element *q*_*j*_(*l*) in **Q** represents the probability that the observation symbol *o*_*l*_ will be represented by the system at time *t*, given that the worker is in state *s*_*j*_ at time *t*. **Q** therefore indicates system false-positive and false-negative effects on the safety risk assessments. *π* = {*π*_*i*_} is the initial state distribution for the worker. Hence, *π*_*i*_ = *P*(*x*_1_ = *s*_*i*_) is the probability that *s*_*i*_ was the initial state of the worker.

As only one hazard is taken into account in the analysis above, (1) can be written as

(4) $$\begin{array}{c} R_{t}^{w}=\overset{M}{\underset{i=1}{\sum }}p_{t}^{w}\left(i\right)\times C\left(i\right), \end{array}$$

where *R*_*t*_^*w*^ is the risk for worker *w* at time *t*, *p*_*t*_^*w*^(*i*) is the probability that the worker is in safety state *s*_*i*_, and *C*(*i*) is the cost value associated with state *s*_*i*_.

Based on the principle of HMM, given an observation symbol *y*_*t*_, and HMM *λ*, the system can update the state probability distribution {*p*_*t*_^*w*^(*i*)} of any worker. The pseudocode updating state probability distribution is as in Pseudocode 1.

**Figure:**
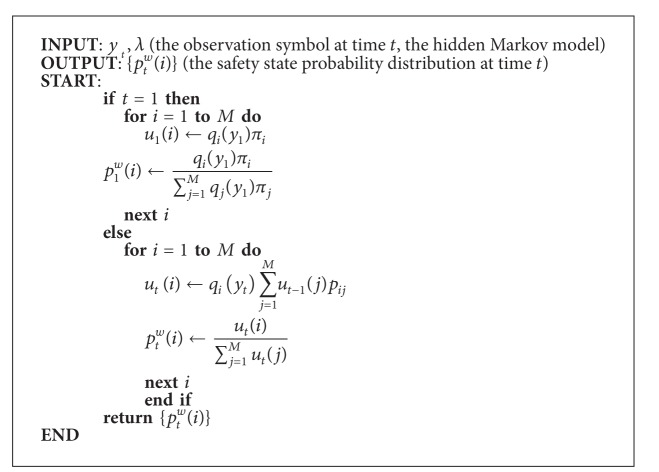
Pseudocode 1

## 3. Simulation Analysis

In order to illustrate the risk assessment method, a simulated analysis was performed. The scope included a worker, a crane (dynamic hazard), a critical edge (static hazard), and a safety supervisor. Positions of the worker, the crane, and the safety supervisor were provided by the RTLS. The position of the critical edge is known and constant. A server regularly received the position data and calculated the relative position relationships between the worker and other assets. The observation symbols indicating safety states and monitoring states were then obtained. For each new symbol, the application used the algorithm in Section 2.3 to update the worker safety states and probabilities and calculated the corresponding risk values. The safety risks associated with the crane and the critical edge and the reduction factor to be applied associated with the safety supervisor were also calculated. Estimating the matrices **P** and **Q**, as well as the cost value *C* and the reduction factor *F* associated with different states, is a nontrivial task outside the scope of this study. The parameters in this case study were chosen qualitatively and only for the purpose of illustration.

### 3.1. Dynamic and Static Hazard Risk

The safety risk associated with the dynamic hazard was first calculated, based on an observation symbol sequence representing the relative positions of workers and the crane. 20 samples were collected using the RTLS and processed by the server. The set *S* = {*N*, *D*, *V*} was used to describe the worker safety state. The observation symbols set is defined as *O* = {*n*, *d*, *v*}, where the symbol *n* indicates state *N*, and so forth. The HMM *λ*_*DY*_ = {**Ρ**_*DY*_, **Q**_*DY*_, *π*_*DY*_} was used, where

(5) ΡDY=(pNNpNDpNVpDNpDDpDVpVNpVDpVV)=(0.9900.0080.0020.1000.8500.0500.0050.0150.980),QDY=(qN(n)qN(d)qN(v)qD(n)qD(d)qD(v)qV(n)qV(d)qV(v))=(0.5000.2500.2500.2500.5500.2000.2000.2000.600),πDY=(πN,πD,πV)=(0.800,0.100,0.100).

**Figure 6:**
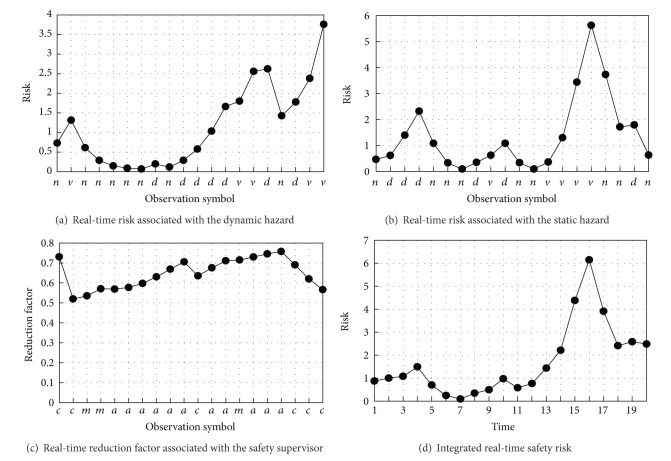
Simulated calculation results illustrating worker safety risk.

Based on the accuracy of GPS and Wi-Fi location technology, the false-positive and false-negative rates were assumed to be relatively high. Hence, values of *q*_*N*_(*d*), *q*_*N*_(*v*), *q*_*D*_(*n*), *q*_*D*_(*v*), *q*_*V*_(*d*), and *q*_*V*_(*n*) are not less than 0.2. The cost value is defined as *C*_*DY*_ = (0,5, 10). Given the observation symbol sequence *Y*, shown on the *x*-axis of Figure 6(a), the dynamic hazard risk is obtained and shown in Figure 6(a).

The static hazard has the same safety state set and observation symbol set as the dynamic hazard. Taking into account the different damage mechanism and different monitored objects, a new HMM *λ*_*ST*_ = {**Ρ**_*ST*_, **Q**_*ST*_, *π*_*ST*_} was created to illustrate static hazard risk assessment:

(6) ΡST=(pNNpNDpNVpDNpDDpDVpVNpVDpVV)=(0.9900.0080.0020.1000.8500.0500.0050.0150.980),QST=(qN(n)qN(d)qN(v)qD(n)qD(d)qD(v)qV(n)qV(d)qV(v))=(0.7000.1500.1500.1500.7500.1000.2000.1500.650),πST=(πN,πD,πV)=(0.800,0.100,0.100).

The main difference is embodied in the fact that the static hazard has lower false-positive and false-negative rates. This is because the static hazard has a fixed and known position which leads to greater accuracy in determining the safety state according to the observation symbol. The cost value is defined as *C*_*ST*_ = (0,3, 7).  Figure 6(b) shows the assessment of the static hazard risk using the HMM *λ*_*ST*_. The observation symbol sequence for the static hazard is not identical to that in Section 3.1, as these are two different kinds of safety state.

### 3.2. Reduction Factor

The reduction factor associated with the presence of the safety supervisor was calculated using the same method. The set *S* = {*C*, *A*, *M*} was used to describe the worker monitor state. *C* (controlled) means that worker behavior was influenced by the supervisor. *A* (affected) indicates a weaker influence than *C*. *M* (missed) means that the worker was not affected by the supervisor. Correspondingly, the observation symbols set is defined as *O* = {*c*, *a*, *m*}, and the HMM *λ*_*RF*_ = {**Ρ**_*RF*_, **Q**_*RF*_, *π*_*RF*_} was used, where

(7) ΡRF=(pCCpCApCMpACpAApAMpMCpMApMM)=(0.9900.0080.0020.1000.8500.0500.0050.0150.980),QRF=(qC(c)qC(a)qC(m)qA(c)qA(a)qA(m)qM(c)qM(a)qM(m))=(0.5000.2500.2500.2500.5500.2000.2000.2000.600),πRF=(πN,πD,πV)=(0.800,0.100,0.100).

The reduction values for the different monitor states were set as *F*_*RF*_ = (0.5,0.8,1). Given the observation symbol sequence *Y* shown on the *x*-axis of Figure 6(c), the reduction factors were obtained from that figure.

### 3.3. Integrated Result

Synthesizing the dynamic hazard risk, the static hazard risk, and the reduction factor, the integrated real-time risk was calculated using (2). The result is shown in Figure 6(d).

To gain an intuitive understanding of the integrated safety risk, the range of the risk values was divided into the 5 levels: low, manageable, elevated, high, and severe, referring to The Code of Construction Project Management, and other literatures [8, 50, 51]. A color-coded metaphor was applied to indicate the five levels of risk severity in this research. From high risk to low, the colors were red, orange, yellow, blue, and green.  Table 2 gives the corresponding relationships.

The safety risk ratings provide the basis for the speed of response. If the risk reaches a certain level, the safety management system may trigger a response to control the risk level. The response may relate to an individual worker or be related to region of the site if there is high risk in a particular zone. An individual-oriented response may be a warning message issued to the workers, or the dispatching of a group of safety supervisors. Examples of a zone-wide response may be a live broadcast alert, a pause of work in the related zone, or even the withdrawal of the workers. These measures, requiring judgment, have to be controlled by management personnel hence provision of the visualization interface and colour-coded risk indicator is important.

## 4. On-Site Application

### 4.1. Setup of the Real-Time Tracking System

The proposed safety risk assessment method was applied to the construction management of the Xiluodu hydropower station, which is the second largest hydropower project in the world generating 13.86 million kW of power after completion [52, 53]. The dam is located on the Jinshajiang River, in Leibo county of Sichuan Province. This high arch dam with a maximum height of 285.5 m is being built by pouring 6.5 million cubic meters of concrete. Thousands of workers are employed and exposed to a complicated working environment. Safety management is a critical concern of the contractors, the supervision company, and the owner. As a result, implementation of the proposed safety control method, especially the establishment of the RTLS, is of great significance. In 2013, infrastructure for GPS and Wi-Fi location facilities was deployed on the dam crest and in all six main tunnels [8].

### 4.2. Site Data Collection

**Figure 7:**
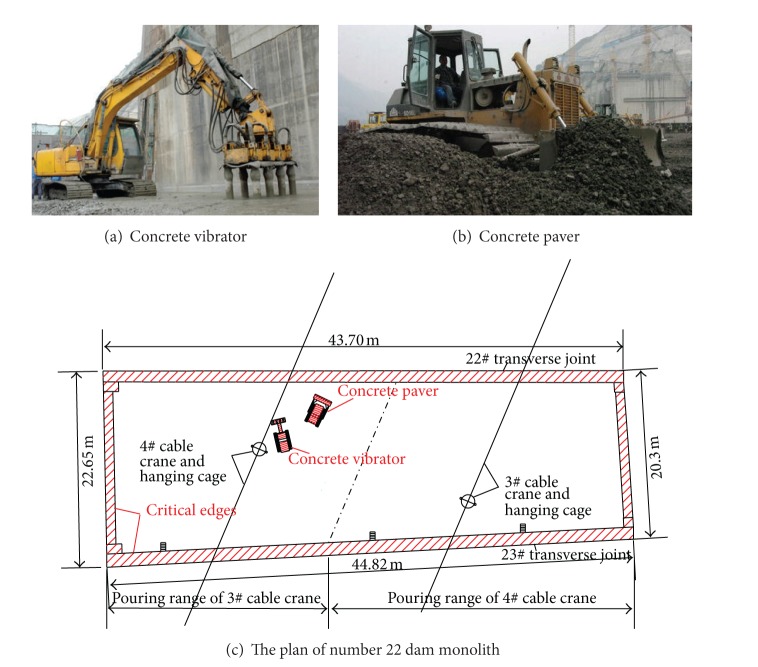
Concrete pouring hazards in number 22 dam monolith.

Site data was collected during the pouring of concrete in number 22 dam monolith. This data consisted of four 20-minute asset trajectories and of a safety supervisor, a concrete vibrator (Figure 7(a)), and a concrete paver (Figure 7(b)), respectively. As the working condition was “outdoors,” the data was collected by GPS model in the smart phone carried by the on-site assets. The geographic coordinates of the trajectories are shown in Table 3. Data was sampled at 10-second intervals. The location information of critical edges in the monolith was obtained from the GIS system for the project. The main dangers to safety came from the two heavy machines (dynamic hazards) and critical edges (static hazards). The number 22 dam monolith plan is shown in Figure 7(c). Both dynamic hazards and static hazards are indicated by the red slashes.

The procedure cycle of the worker is detailed as follows.

**Step 1 .**

Move to the concrete pouring area from the edge of the dam monolith.

**Step 2 .**

Catch the hanging cage delivered by the cable crane and open the cage to release the concrete.

**Step 3 .**

Move back to the edges and wait for the concrete paver and the concrete vibrator to complete their work on the fresh concrete.

**Step 4 .**

Pave and vibrate the concrete at the edges and corners using manual labour.

**Step 5 .**

Wait for the hanging cage to arrive again at the pouring area.

### 4.3. Results and Discussions

**Figure 8:**
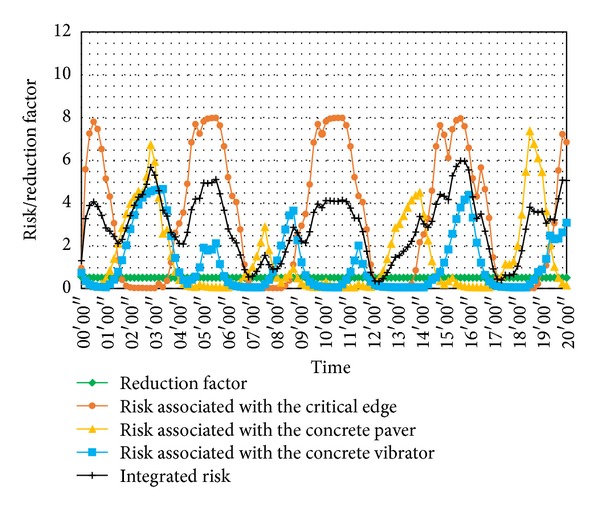
Real-time safety risks to a worker situated in the pouring area for a 20-minute period.

The HMM parameters *λ*_*DY*_, *λ*_*ST*_, and *λ*_*RF*_ used in this calculation were the same as those of the simulation in Section 3 as well as the safety state sets and the observation symbol sets. The cost and supervisor reduction values and the distances involved related to the different states are given in Table 4. The worker safety risk assessment results in the 20 minutes concerned are shown in Figure 8. Variation for each risk, the reduction factor, and the integrated risk are presented in different colors.

From Figure 8, periodic changes occur in worker safety risks, as the working procedure is cyclical. The cycle time is controlled by the cable crane cycle of about 5 minutes in delivering the concrete bucket. The most obvious periodic risk is associated with the critical edge. Five peaks in the orange line are evident in Figure 8, as workers approached and entered the critical edge zone on 5 occasions. Focusing on the yellow and blue lines, risks associated with the two heavy machines also clearly fluctuated. That was because the paving and vibrating procedures caused the machines to intermittently approach the workers. The green line indicating the reduction factor resulting from the presence of the safety supervisor remains constant, which means that the safety supervisor was strictly monitoring the workers throughout the task. The integrated risk is represented by the black line. Peak values of the integrated risk appear at 2′50′′, 5′30′′, 8′40′′, 11′00′′, 15′30′′, and 19′10′′. At those moments, the workers walked back to the critical edge and manually paved or vibrated the concrete, while the machines were busy with the fresh concrete which might approach the workers from time to time. There was, therefore, the superposition of several risks. From field data based calculations, it can be concluded that the safety supervisor as well as the worker should pay particular attention when manual paving and vibrating are occurring at the critical edges.

In conclusion, the on-site application results are in line with the actual site situation. Dangerous states caused by worker behavior, the hazards on-site, and the effect of safety supervisor presence can be embodied in the risk value.

## 5. Conclusions

A RTSRA method has been presented in this paper. The method based on the HMM processes the location data obtained from the RTLS and gives the engineers and project managers real-time safety risk assessments applying to on-site workers. The following conclusions can be drawn.

1. The RTSRA implements a quantitative, Human-Centered, and real-time safety risk assessment. Factors related to the real-time safety risk of an on-site worker have been classified and quantified. The real-time safety risk values and reduction factors are obtained using a proposed reliable formula for quantifying risks. Based on the HMM, the RTSRA gives the real-time probability distributions of different safety states/monitor states and subsequent safety risk values.
2. When combined with the real-time location system, the RTSRA builds a logical relationship between safety risk and the locations of on-site assets. The proposed method leverages the massive data produced by the RTLS to provide more abundant site information. Such information enables project managers and engineers to anticipate the locality and potential occurrence of safety risk and contributes to more effective computerized decision processes.
3. The on-site application shows that the proposed method is reliable and effective for real-time safety risk assessment. The logic and the function of the proposed novel method fulfill the goals of safety management.

The RTSRA method was broadly applied to the safety management of a single concrete poured on the Xiluodu dam hydropower construction site. Further study, however, is necessary to focus on case statistics and optimization of the HMM parameters concerned in improving the accuracy and effectiveness of the safety risk assessment method proposed.

## Figures and Tables

**Table 1:** Dynamic and static hazards and their associated activities on construction sites.

	Hazards description	Associated activity
Dynamic hazards	(1) Collision with or entrapment by a moving load due to its movement or detachment	Mechanical load handling.
	(2) Collision with or being ran over by heavy equipment or heavy goods vehicles	Work with heavy equipment or heavy-goods vehicles
	(3) Cuts, blunt trauma, and other injuries due to light equipment	Work with light equipment
	(4) Burns	Welding
	(5) Injury due to the impact of falling objects and projectiles	Manual, mechanical, or explosive demolition; shot-hole drilling before the blasting of a cut slope and the subsequent cleanup and field survey
	(6) Collision with or being ran over by vehicles unrelated to the construction work	Work in areas with traffic unrelated to construction work
	(7) Traffic accident	Transport of equipment and materials to the construction site

Static hazards	(1) Fall to lower levels	Work at heights or depths of more than 2 m
	(2) Direct or indirect electrical contact	Electrical work, work in proximity to power lines, and work with electrical equipment under wet conditions
	(3) Burns caused by fire or explosion due to a ruptured pipeline	Work close to fuel pipelines
	(4) Gas inhalation	Work near gas pipelines
	(5) Entrapment and subsequent suffocation due to a landslide	earthmoving, excavation, shafts, underground work, and tunnels
	(6) Particle projection and accidental explosion	Blasting for excavation, shafts, underground work, and tunnels
	(7) Decompression sickness	Work under hyperbaric conditions
	(8) Blows to upper and lower limbs	Manual load handling
	(9) Acute dust and toxin poisoning	Manual, mechanical, or explosive demolition of structures or buildings in general and of hospitals, factories, slaughterhouses, or any other place that may contain toxic substances
	(10) Suffocation or poisoning in confined spaces	Work in confined spaces
	(11) Drowning	Work in areas at risk of flooding
	(12) Structural risk or macrorisk	Complex operations or structures

**Table 2:** Qualitative and numerical ratings for the risk value of a worker.

Risk value	Qualitative description	Color
>8	Severe	Red
6–8	High	Orange
4–6	Elevated	Yellow
2–4	Manageable	Blue
<2	Low	Green

**Table 3:** Records of the trajectories.

RECORD_ID	ASSET_ID	COORD_*X*	COORD_*Y*	SYSTEM_TIME
2316426	43	103.6511012	28.2600214	16:05:03
2316427	43	103.6511090	28.2600114	16:05:13
2316428	43	103.6511015	28.2600036	16:05:23
2316429	43	103.6511142	28.2600182	16:05:33
2316426	43	103.6511355	28.2601391	16:05:43
⋮	⋮	⋮	⋮	⋮
2317866	69	103.6508376	28.2601321	17:05:03
2317867	69	103.6508376	28.2599679	17:05:04

**Table 4:** Parameters for the safety risk assessment.

Item	Cost/reduction value ((*N*, *D*,* V*)/(*C*,* A*,* M*))	Distance for very dangerous/controlled	Distance for dangerous/affected	Distance for normal/missed
Critical edge	(0, 4, 8)	<1 m	1 m-2 m	>2 m
Concrete vibrator	(0, 5, 10)	<3 m	3 m–5 m	>5 m
Concrete paver	(0, 5, 10)	<3 m	3 m–5 m	>5 m
Safety supervisor	(0.5, 0.8, 1)	<5 m	5 m–10 m	>10 m
